# Supplementary material for: HIVprotI: an integrated web based platform for prediction and design of HIV proteins inhibitors
Source: J Cheminform. 2018 Mar 9;10:12. doi: 10.1186/s13321-018-0266-y (PMC5845081; doi:10.1186/s13321-018-0266-y)
Supplement: Supplementary file 2 — Additional file 2. Source code of HIVProtI web server. [file 13321_2018_266_MOESM2_ESM.zip › HIVprotI_Source-code/hivprotI/weka-3-6-6/doc/weka/gui/beans/TestSetProducer.html]

TestSetProducer


---


|  |  |  |
| --- | --- | --- |
| **Overview** \| **Package** \| **Class** \| **Tree** \| **Deprecated** \| **Index** \| **Help** \| **Weka's home** | |  |
| **PREV CLASS**   **NEXT CLASS** | **FRAMES**    **NO FRAMES**     **All Classes** |  |
| SUMMARY: NESTED \| FIELD \| CONSTR \| METHOD | DETAIL: FIELD \| CONSTR \| METHOD |  |


---


## weka.gui.beans Interface TestSetProducer

**All Known Implementing Classes:**: AbstractTestSetProducer, AbstractTrainAndTestSetProducer, ClassAssigner, CrossValidationFoldMaker, Filter, PredictionAppender, TestSetMaker, TrainTestSplitMaker

---

``` public interface TestSetProducer ```

Interface to something that can produce test sets

**Version:**
:   $Revision: 1.3 $

**Author:**
:   Mark Hall

---

| **Method Summary** | |
| --- | --- |
| `void` | `addTestSetListener(TestSetListener tsl)`             Add a listener for test set events |
| `void` | `removeTestSetListener(TestSetListener tsl)`             Remove a listener for test set events |

**Method Detail**

### addTestSetListener

```
void addTestSetListener(TestSetListener tsl)
```

:   Add a listener for test set events

    :   **Parameters:**: `tsl` - a `TestSetListener` value

---


### removeTestSetListener

```
void removeTestSetListener(TestSetListener tsl)
```

:   Remove a listener for test set events

    :   **Parameters:**: `tsl` - a `TestSetListener` value


---


|  |  |  |
| --- | --- | --- |
| **Overview** \| **Package** \| **Class** \| **Tree** \| **Deprecated** \| **Index** \| **Help** \| **Weka's home** | |  |
| **PREV CLASS**   **NEXT CLASS** | **FRAMES**    **NO FRAMES**     **All Classes** |  |
| SUMMARY: NESTED \| FIELD \| CONSTR \| METHOD | DETAIL: FIELD \| CONSTR \| METHOD |  |


---
